# Renal IL-18 Production Is Macrophage Independent During Obstructive Injury

**DOI:** 10.1371/journal.pone.0047417

**Published:** 2012-10-12

**Authors:** Ethan I. Franke, Brian A. Vanderbrink, Karen L. Hile, Hongji Zhang, Alexandra Cain, Futoshi Matsui, Kirstan K. Meldrum

**Affiliations:** 1 Department of Urology, Indiana University School of Medicine, Indianapolis, Indiana, United States of America; 2 Department of Urology, University of Florida, Gainesville, Florida, United States of America; Institut National de la Santé et de la Recherche Médicale, France

## Abstract

**Background:**

Interleukin 18 (IL-18) is a pro-inflammatory cytokine that mediates fibrotic renal injury during obstruction. Macrophages are a well-known source of IL-18; however, renal tubular epithelial cells are also a potential source of this cytokine. We hypothesized that IL-18 is predominantly a renal tubular cell product and is produced during renal obstruction independent of macrophage infiltration.

**Methods:**

To study this, male C57BL6 mice were subjected to unilateral ureteral obstruction (UUO) vs. sham operation in the presence or absence of macrophage depletion (liposomal clodronate (1 ml/100 g body weight i.v.)). The animals were sacrificed 1 week after surgery and renal cortical tissue harvested. Tissue levels of active IL-18 (ELISA), IL-18 receptor mRNA expression (real time PCR), and active caspase-1 expression (western blot) were measured. The cellular localization of IL-18 and IL-18R was assessed using dual labeling immunofluorescent staining (IFS).

**Results:**

Immunohistochemical staining of renal tissue sections confirmed macrophage depletion by liposomal clodronate. IL-18 production, IL-18R expression, and active caspase 1 expression were elevated in response to renal obstruction and did not decline to a significant degree in the presence of macrophage depletion. Obstruction-induced IL-18 and IL-18R production localized predominantly to tubular epithelial cells (TEC) during obstruction despite macrophage depletion.

**Conclusion:**

These results demonstrate that renal tubular epithelial cells are the primary source of IL-18 production during obstructive injury, and that tubular cell production of IL-18 occurs independent of macrophage infiltration.

## Introduction

Upper urinary tract obstruction results in a progressive, and eventually, permanent, loss in renal function as a result of apoptotic cell death and severe tubulointerstitial fibrosis. The pathophysiology of obstructive nephropathy is a complex process, involving inflammatory cell infiltration, fibroblast proliferation, and an imbalance in extracellular matrix (ECM) synthesis, deposition, and degradation [1]. Interstitial inflammatory cell infiltration occurs shortly after the onset of renal obstruction [1], [2] and results in the release of a variety of cytokines and growth factors that stimulate ECM synthesis and fibroblast proliferation. Interleukin 18 (IL-18) is a pro-inflammatory cytokine that has been implicated in the pathogenesis of many inflammatory renal disease, including renal ischemia-reperfusion injury, allograft rejection, autoimmune disease, and recently, obstructive uropathy [3]–[7].

Interleukin-18 (IL-18) is a pro-inflammatory cytokine that is structurally and functionally related to the IL-1 family, and a potent stimulator of cytokine gene expression and synthesis via activation of NF**κ**B. IL-18 is synthesized as a biologically inactive precursor (pro-IL-18), similar to IL-1β, which requires cleavage into an active molecule by the intracellular cysteine protease IL-1β converting enzyme (ICE) or caspase-1 [8]. Caspase-1 is remarkably specific for the precursors of IL-1β and IL-18, making a single initial cut in each pro-cytokine that results in the extracellular secretion of active mature cytokine. [9], [10]. While IL-18 is largely regarded as a macrophage product, we have recently demonstrated that tubular epithelial cells are an important source of renal IL-18 production during obstruction [11]. We therefore hypothesized that renal IL-18 production during obstruction is a macrophage independent process. To study this, we examined renal cortical macrophage infiltration, IL-18 production, IL-18R expression, caspase 1 expression, and the cellular localization of IL-18 and IL-18R production in male C57B16 mice exposed to macrophage depletion or control conditions using a well established model of unilateral ureteral obstruction (UUO).

## Materials and Methods

### Animals, experimental groups, and operative techniques

The animal protocol was reviewed and accepted by the Animal Care and Research Committee of the Indiana University School of Medicine. Macrophage depletion was achieved by tail vein injection of liposomal clodronate (1 ml/100 g body weight). Two-month old male wild-type C57BL6 mice were exposed to liposomal clodronate or control liposomes 48 hours prior to surgery. On day 2, mice were anesthetized with isofluorane and subjected to either sham operation or left UUO (5 animals per group). For obstructed animals, the left ureter was isolated and completely ligated with 5–0 silk suture. Sham-operated mice underwent an identical surgical procedure without ureteral ligation. Liposomal clodronate vs. control liposomes were delivered at the time of surgery and every 48 hours thereafter until the animals were sacrificed. After one week, the mice were re-anesthetized, the left kidneys removed and snap frozen in liquid nitrogen, and the animals subsequently sacrificed.

### Macrophage Accumulation

Transverse 4 mm renal tissue sections were deparaffinized and dehydrated with xylene and alcohol. Antigen was retrieved by incubating the cells with proteinase K for 20 min in an oven. The tissues were then blocked with 1% bovine serum albumin (BSA) and incubated with a a rat anti-mouse macrophage antibody (F4/80; 1∶100, Angio-Proteomie, Boston, MA) for 90 min at room temperature. Sections were washed and incubated with a secondary antibody (goat anti-rat (1∶100)) and incubated for 30 minutes. Peroxidase-stained sections were then developed with 3,3″-diaminobenzidine (DAB) and counterstained with hematoxylin (Sigma-Aldrich). Sections incubated without primary antibody exhibited no staining. Sections from each sample were then photographed (X400) and analyzed for macrophage accumulation.

### Tissue homogenization

A portion of each renal cortex was homogenized after the samples had been diluted in 10 volumes of homogenate buffer per gram of tissue (10 mM Hepes (pH 7.9), 10 mM KCL, 0.1 mM EGTA, 1 mM DTT, and Complete Protease Inhibitor tabs (Boehringer Mannheim, Indianapolis, IN, USA)) using a vertishear tissue homogenizer. Renal homogenates were then centrifuged at 3000×*g* for 15 min at 4°C, and the supernatants stored at −80°C until the ELISA assays or western blots could be performed.

### Active IL-18 ELISA

Renal homogenate active IL-18 levels were determined using an ELISA (mouse IL-18: MBL Int., Woburn, MA, USA). The ELISA was performed by adding 100 µl of each sample to wells in a 96-well plate of a commercially available ELISA kit and the assay performed according to the manufacturer's instructions. All samples were tested in duplicate. The ELISA results were expressed as pg/mg protein.

### Real-time PCR

Total RNA was extracted from renal cortical tissue by homogenization in Trizol (Gibco BRL, Gaithersburg, MD, USA), then isolated by precipitation with chloroform and isopropanol. Total RNA (0.5 mg) was subjected to cDNA synthesis using iScript (Bio-Rad, Hercules, CA, USA). cDNA from each sample was analyzed for IL-18 receptor (Mm00515180_m1) using a TaqMan gene expression assay (RT-PCR; Applied Biosystems, Foster City, CA, USA). FAM Dye/MGB-labeled probes for mouse β-actin (Applied Biosystems) served as endogenous controls.

### Western Blot Analysis

Protein extracts from homogenized samples (30 µg per lane) were subjected to SDS-PAGE electrophoresis on a Tris-Glycine gel and transferred to a polyvinylidene fluoride membrane. Immunoblotting was performed by incubating each membrane in 5% dry milk for 1 hour, followed by incubation with an anti-caspase-1 p20 antibody (1∶200 overnight at 4°C, MBL International Corp, Woburn, MA). After being washed three times in TBST, each membrane was incubated for 1 hour at RT with a peroxidase-conjugated secondary antibody (1∶3,000, Thermo Scientific, Rockford, IL). Equivalent protein loading in each lane was confirmed by stripping and re-blotting each membrane for GAPDH (1∶20,000 for 30 min at RT, secondary 1∶20,000 for 30 min at RT; Biodesign International, Saco, ME, USA). The membranes were developed using enhanced chemiluminescence (Amersham Pharmacia Biotech Inc., Piscataway, NJ, USA), and the density of each band determined using NIH image analysis software and expressed as a percentage of GAPDH density.

### Cellular Immunolocalization

Five micrometer renal sections were harvested from each sample, fixed in acetone for 10 minutes, rinsed in phosphate buffered solution (PBS) three times, and incubated for 20 minutes in blocking solution containing 10% goat or donkey serum. Slides were then incubated with goat anti-IL-18 (1∶50; Santa Cruz Biotechnology; Santa Cruz, CA) or rabbit anti-IL-18R (1∶50, Santa Cruz Biotechnology; Santa Cruz, CA) for 60 min at room temperature. Slides were then washed three times in PBS and incubated with a secondary antibody for 45 minutes at room temperature (donkey anti-goat Texas Red IgG 1∶200 or donkey anti-rabbit Texas Red IgG 1∶200 (Santa Cruz Biotechnology; Santa Cruz, CA)). The distal renal tubules were counterstained fluorescein Peanut Agglutinin for 15 minutes (5 mg/ml; 1∶500 in PBS with 1% Goat Serum; Vector Laboratories). The nuclei were then stained with bis-Benzimide (10 mg/ml). To assess the specificity of the immunostaining, adjacent sections were incubated without primary antibody and processed using identical conditions. The slides were mounted and stored at −4°C. Digital photographs were taken (400X) using a fluorescent microscope (Leica DM IRB; Wetzlar, Germany).

### Statistical Analysis

Data are presented as means±SD. Differences at the 95% confidence intervals were considered significant. The experimental groups were compared using ANOVA with post hoc Bonferroni-Dunn (JMP Statistical Software version 5.0; Berkeley, CA).

## Results

### Macrophage Infiltration

**Figure 1:**
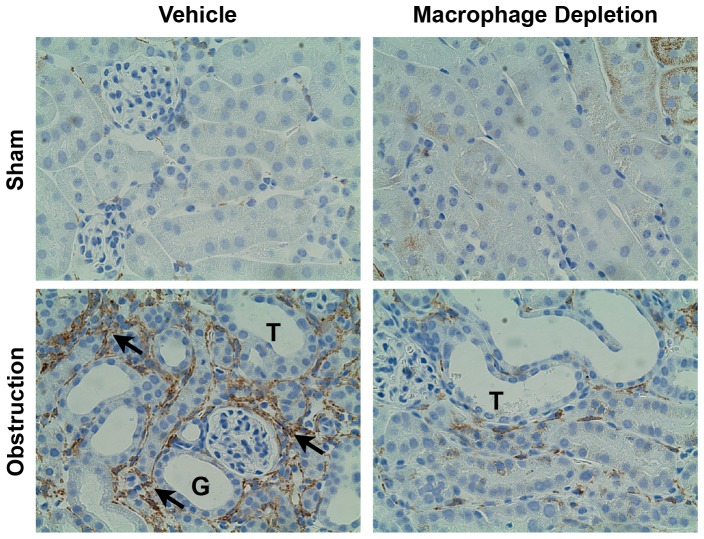
Renal cortical macrophage accumulation following UUO. Photographs (magnification 400X) depicting macrophage (brown stain; arrows) in vehicle treated and macrophage depleted mice exposed to sham operation or one week of UUO. T = tubule; G = glomerulus.

Renal cortical tissue sections were stained with a rat anti-mouse macrophage antibody to assess the degree of macrophage infiltration during renal obstruction. Although sham-treated samples exhibited minimal macrophage staining (Figure 1), mice subjected to one week of renal obstruction demonstrated significant interstitial macrophage accumulation. The degree of obstruction-induced macrophage infiltration was significantly reduced in the presence of liposomal clodronate, thereby demonstrating effective macrophage depletion in this animal model.

### Renal IL-18 Production and IL-18 Receptor expression

**Figure 2:**
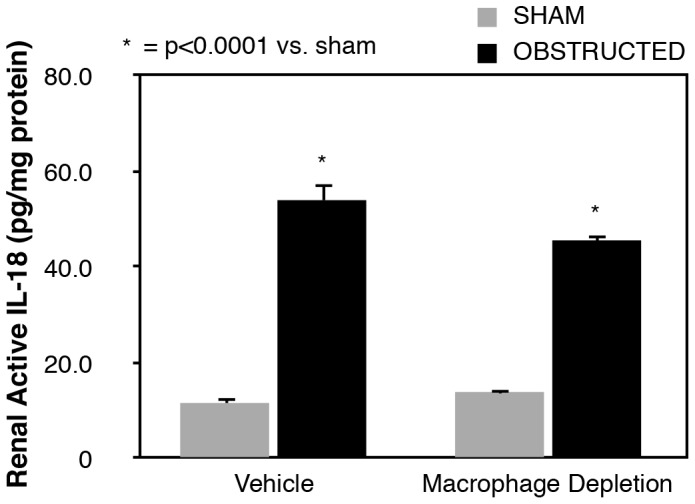
Renal cortical active IL-18 production following UUO. Renal cortical active IL-18 protein levels in vehicle treated and macrophage depleted mice exposed to sham operation or one week of UUO.

Active renal IL-18 production remained low in sham treated animals, but increased significantly in response to one week of obstruction (Figure 2). The increase in obstruction-induced IL-18 production persisted despite macrophage depletion, with no significant difference in IL-18 levels observed between vehicle treated and macrophage depleted animals.

**Figure 3:**
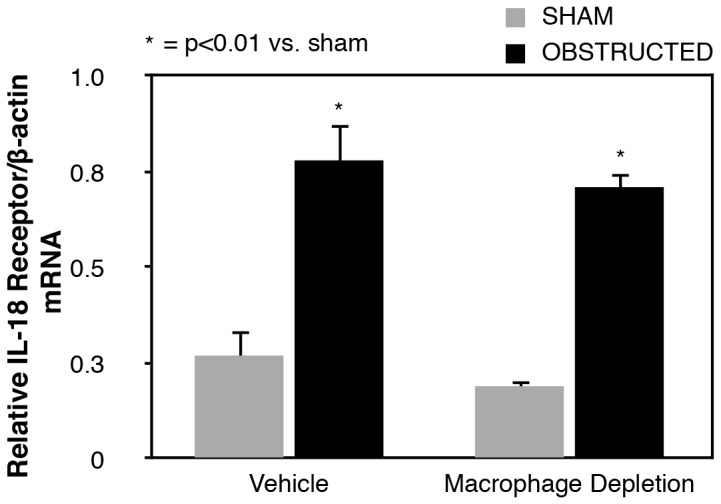
Renal cortical IL-18 receptor mRNA expression following UUO. Quantitative IL-18 receptor (IL-18R) mRNA expression represented as a percentage of β-actin in vehicle treated and macrophage depleted mice exposed to sham operation or one week of UUO.

Similarly, renal IL-18R mRNA expression increased significantly in response to renal obstruction (Figure 3) in both vehicle treated and macrophage depleted animals as compared to shams. No significant difference in the level of obstruction-induced renal IL-18R mRNA expression was detected between vehicle treated and macrophage depleted animals.

### Caspase-1 activation

**Figure 4:**
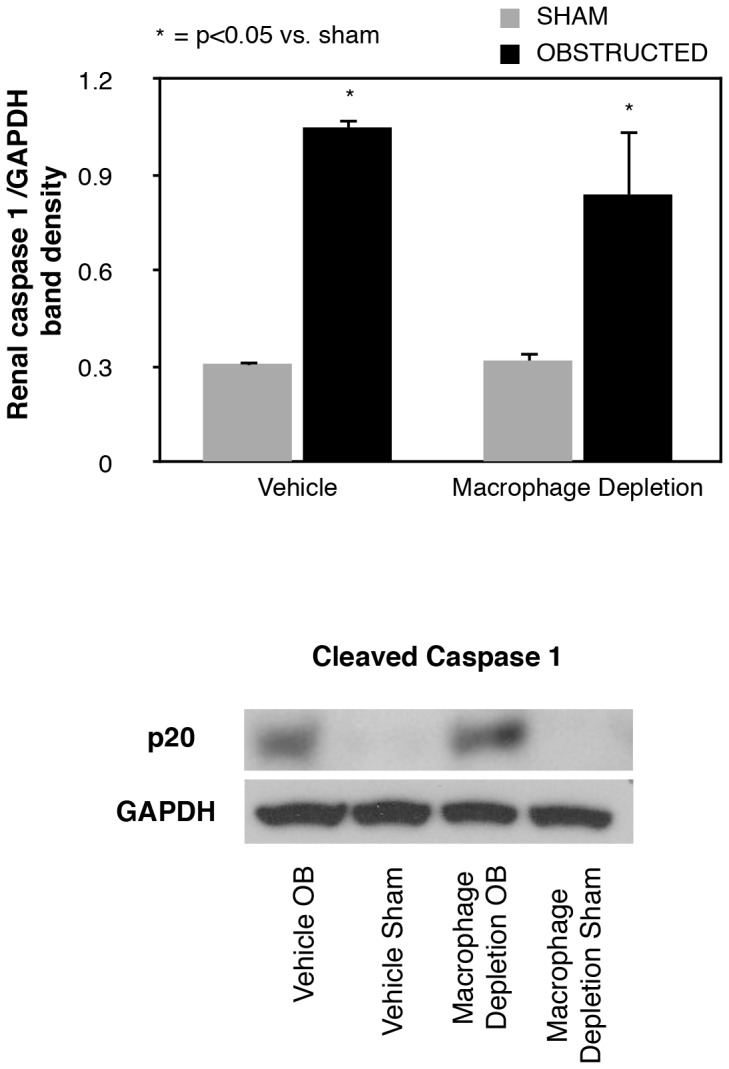
Renal cortical active caspase-1 expression following UUO. Gel photograph and densitometric analysis of active caspase-1 expression represented as a percentage of GAPDH in vehicle treated and macrophage depleted mice exposed to sham operation or one week of UUO.

Caspase 1 expression was then evaluated to determine the impact of macrophage depletion on the mechanism of IL-18 activation. Active caspase 1 expression increased significantly in response to renal obstruction (Figure 4) in both vehicle treated and macrophage depleted animals as compared to shams. No significant difference in the caspase 1 expression was detected between vehicle treated and macrophage depleted animals.

### IL-18 & IL-18 Receptor Localization

**Figure 5:**
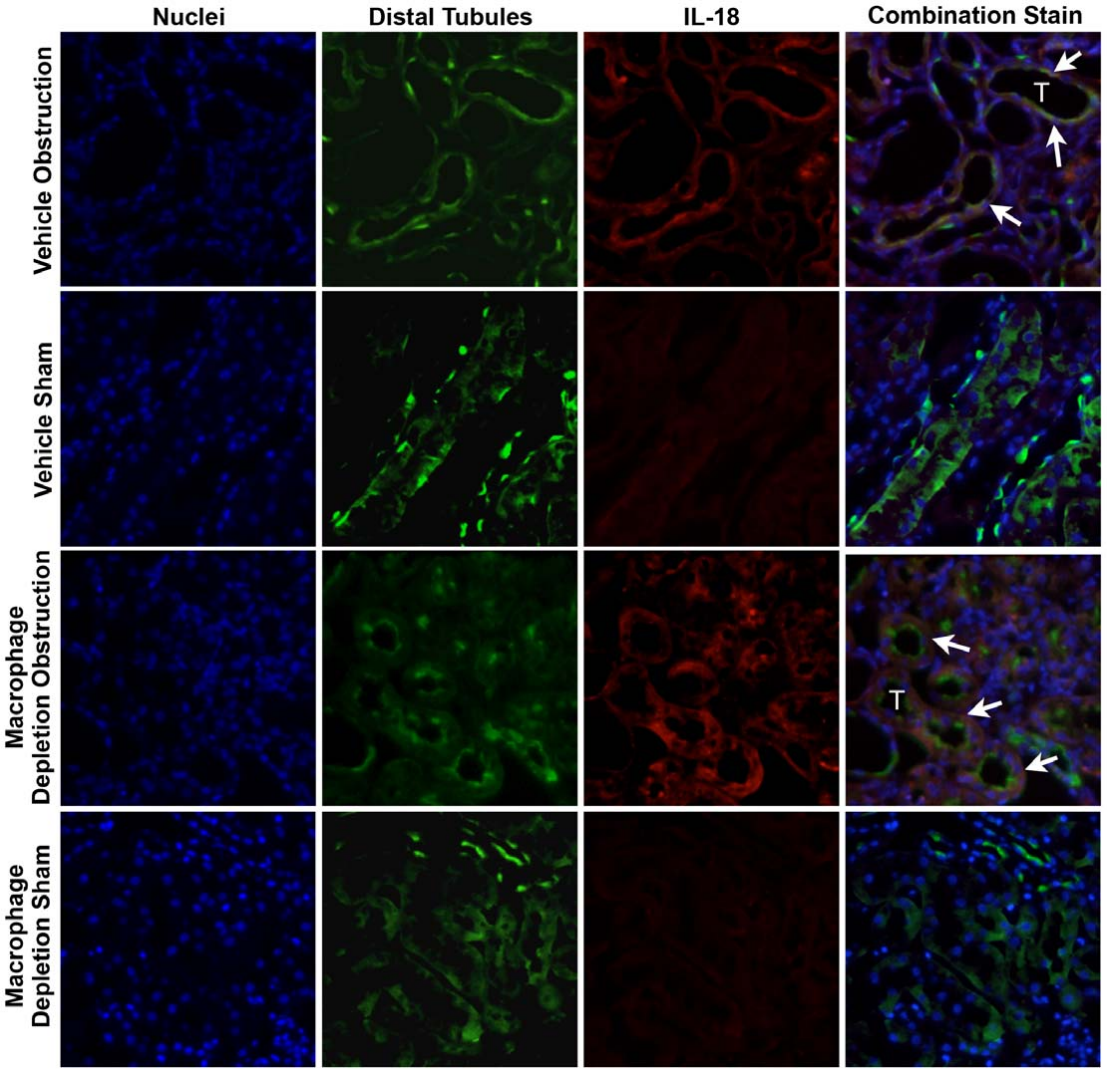
Renal cortical dual labeled immunofluorescent staining for IL-18 and tubular epithelial cells following UUO. **A.** Photographs (magnification 400X) depicting renal cortical IL-18 production (red) and distal tubular staining (Peanut Agglutinin; green) in vehicle treated and macrophage depleted mice exposed to sham operation or one week of UUO. White arrows indicate IL-18 staining overlying tubules. T = Tubules.

**Figure 6:**
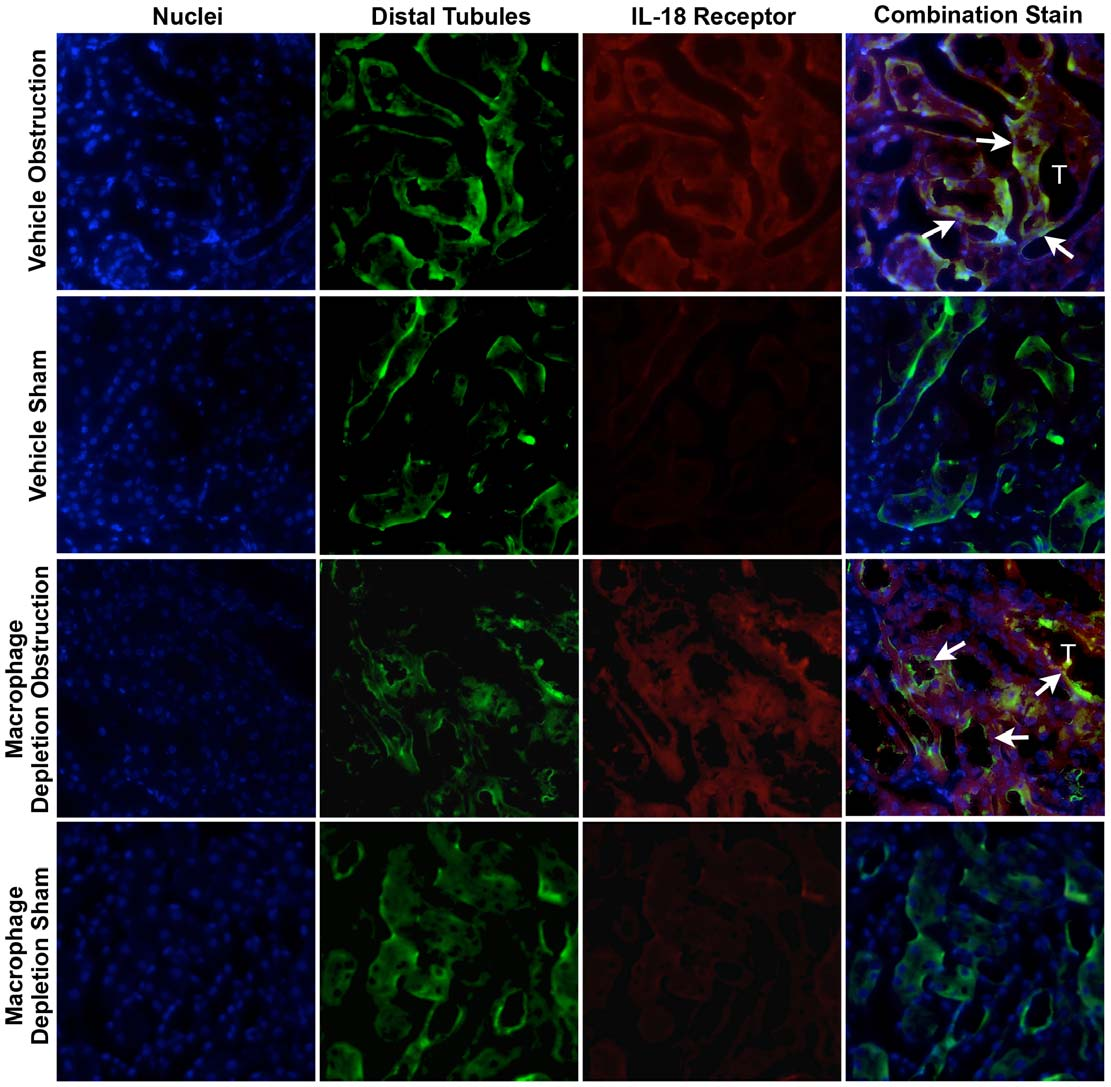
Renal cortical dual labeled immunofluorescent staining for IL-18R and tubular epithelial cells following UUO. **A.** Photographs (magnification 400X) depicting renal cortical IL-18R production (red) and distal tubular staining (Peanut Agglutinin; green) in vehicle treated and macrophage depleted mice exposed to sham operation or one week of UUO. White arrows indicate IL-18R staining overlying tubules. T = Tubules.

The cellular localization of IL-18 and IL-18R was determined using dual labeling immunofluorescent staining. Results demonstrate a significant increase in IL-18 (Figure 5) and IL-18R (Figure 6) staining in the kidneys of both vehicle treated and macrophage depleted animals as compared to shams. IL-18 and IL-18R production localized predominantly to tubular epithelial cells (TEC), and to a much lesser extent the renal interstitium. The degree of IL-18 and IL-18R staining was not noticeably different between vehicle treated and macrophage depleted animals.

## Discussion

Obstructive renal injury is a complex physiological process resulting in tubular dilation, progressive tubulointerstitial fibrosis, apoptotic cell death, and eventual renal dysfunction. Macrophage infiltration occurs shortly after the onset of renal obstruction [1], [2] and results in the release of a variety of cytokines and growth factors that stimulate ECM synthesis and fibroblast proliferation. IL-18 has recently been identified as an important mediator of obstructive renal injury, stimulating both tubulointerstitial fibrosis and tubular epithelial cell apoptosis during obstruction independent of TGF-β1 production [7], [12]. While IL-18 is widely recognized as a macrophage product, IL-18 can be produced from a wide variety of cells in an inactive state. We have recently demonstrated that renal tubular cells produce IL-18 during obstructive injury[11], [13], and therefore hypothesized that obstruction-induced renal IL-18 production is a macrophage independent process. This is the first study to demonstrate that IL-18 production, IL-18R expression, and active caspase 1 expression occur independent of macrophage infiltration during renal obstruction, and that IL-18 and IL-18R production localize primarily to tubular epithelial cells in both vehicle treated and macrophage depleted animals.

While macrophage are a well known immunologic source of IL-18 [14], accumulating evidence suggests that renal tubular epithelial cells are an important source of IL-18 production during fibrotic renal injury [7], [11], [15], [16]. Our data reveal that renal active IL-18 production and IL-18 receptor expression are significantly elevated in response to obstruction despite macrophage depletion, and further, that IL-18 and IL-18R mRNA levels are not significantly different between vehicle treated and macrophage depleted animals. Since IL-18′s activity is dependent on processing by caspase 1, an enzyme that converts inactive pro-IL-18 into active, mature cytokine [8], we further examined the impact of macrophage depletion on active caspase 1 expression. As expected, caspase 1 expression is significantly elevated in response to obstruction. Active caspase 1 expression; however, remains significantly elevated in response to obstruction despite macrophage depletion, and expression levels are not significantly different between vehicle treated and macrophage depleted animals. These results cumulatively suggest that IL-18 production, the mechanism for IL-18 activation (caspase 1), and the mechanism for IL-18 signal transduction (IL-18R), are macrophage independent processes during obstructive renal injury.

In order to specifically localize cellular production of IL-18 and IL-18R during obstructive renal injury, dual labeling immunofluorescent studies were performed. Both IL-18 and IL-18R production localized predominantly to tubular epithelial cells during renal obstruction. Animals subjected to macrophage depletion showed a similar localization pattern in response to obstruction, and no appreciable difference in the degree of staining for IL-18 or IL-18R was detected between vehicle treated and macrophage depleted animals. This data provides further evidence that IL-18 production is macrophage independent during obstructive renal injury, and further, identifies tubular epithelial cells as the predominant site of both IL-18 and IL-18R production during renal obstruction. This finding is supported by observations from He et al [17], who demonstrated that renal IL-18 production was not diminished in macrophage depleted mice in response to ischemia, and further, that the adoptive transfer of peritoneal macrophages with inhibited IL-18 function did not reverse the functional protection afforded by macrophage depletion. This observation led the authors to suggest that tubular cells, not macrophages, are the primary source of increased IL-18 during ischemic renal injury [17].

## Conclusions

Renal obstruction stimulates a signaling cascade that results in IL-18 production and subsequent renal injury. While macrophage infiltration is an early event in obstructive renal injury and macrophage are well known immunologic source of IL-18, this study clearly demonstrates that IL-18 production and the mechanisms of IL-18 activation and IL-18 signal transduction are macrophage independent processes during renal obstruction. This study further demonstrates that the predominant source of both IL-18 and IL-18R during renal obstruction is the tubular epithelial cell. As the mechanisms and localization of renal IL-18 production become more clearly defined, therapeutic strategies aimed at ameliorating obstruction-induced renal injury may be realized.
